# Chemotherapy-Induced Tumor Cell Death at the Crossroads Between Immunogenicity and Immunotolerance: Focus on Acute Myeloid Leukemia

**DOI:** 10.3389/fonc.2019.01004

**Published:** 2019-10-09

**Authors:** Darina Ocadlikova, Mariangela Lecciso, Alessandro Isidori, Federica Loscocco, Giuseppe Visani, Sergio Amadori, Michele Cavo, Antonio Curti

**Affiliations:** ^1^Department of Hematology and Oncology, University Hospital S.Orsola-Malpighi, Institute of Hematology “L. and A. Seràgnoli”, Bologna, Italy; ^2^Hematology and Stem Cell Transplant Center, AORMN Hospital, Pesaro, Italy; ^3^Department of Medicine, Institute of Hematology, University Hospital Tor Vergata, Rome, Italy

**Keywords:** acute myeloid leukemia, immunogenic cell death, dendritic cells, T regulatory cells, immunosuppression

## Abstract

In solid tumors and hematological malignancies, including acute myeloid leukemia, some chemotherapeutic agents, such as anthracyclines, have proven to activate an immune response via dendritic cell-based cross-priming of anti-tumor T lymphocytes. This process, known as immunogenic cell death, is characterized by a variety of tumor cell modifications, i.e., cell surface translocation of calreticulin, extracellular release of adenosine triphosphate and pro-inflammatory factors, such as high mobility group box 1 proteins. However, in addition to with immunogenic cell death, chemotherapy is known to induce inflammatory modifications within the tumor microenvironment, which may also elicit immunosuppressive pathways. In particular, DCs may be driven to acquire tolerogenic features, such as the overexpression of indoleamine 2,3-dioxygensase 1, which may ultimately hamper anti-tumor T-cells via the induction of T regulatory cells. The aim of this review is to summarize the current knowledge about the mechanisms and effects by which chemotherapy results in both activation and suppression of anti-tumor immune response. Indeed, a better understanding of the whole process underlying chemotherapy-induced alterations of the immunological tumor microenvironment has important clinical implications to fully exploit the immunogenic potential of anti-leukemia agents and tune their application.

## Acute Myeloid Leukemia

Acute myeloid leukemia (AML) is a clonal disorder sprouting from a rare population of leukemic stem cells with impaired differentiation capacity into fully mature myelocytic cells. Although new and potent drugs have recently entered the clinical stage, the induction therapy of AML is still principally based on cytotoxic drugs which are able to achieve complete remission (CR) in up to 70% of adult patients (1, 2). However, the probability of relapse remains elevated, in particular in elderly or prognostically “high risk” patients, unless transplantation of autologous or, more importantly, allogeneic hematopoietic stem cells is performed as post-CR consolidation strategy (3).

In the last few years, cancer immunotherapy, which is based on the ability of the immune system to recognize tumor-associated antigens (TAAs) and mediate a highly specific cytolytic response against tumor cells, is gaining much interest due to its unique characteristics, such as the absence of conventional drug resistance mechanisms and low grade of toxicity. In AML, the immunotherapy field is evolving and expanding. In this scenario, recent promising clinical results support the full development of immune-based strategies for the management of AML patients.

## Immunogenicity of AML and Immunotherapy

The data demonstrating increased incidence of solid tumors in immune-compromised patients, spontaneous immune-based regression of some tumors and favorable prognostic impact of tumor-infiltrating cytotoxic T lymphocytes (CTLs) or serum tumor-specific antibodies support the hypothesis that the immune system plays a very important role in tumor development, growth, and progression (4). The most clear demonstration of tumor eradication by the immune system comes from the setting of hematopoietic stem cell transplantation (SCT), where the existence of the Graft vs. Leukemia effect accounts for the prominent therapeutic activity of transplantation (5).

AML is a neoplasm with characteristics which make it suitable to elicit effective specific immune responses. Indeed, 50–90% of cases reveal chromosomal anomalies, above all translocations, which give rise to rejected tumor antigens, namely neo-antigens, not expressed by normal cells. Moreover, leukemia cells express elevated levels of TAAs, which can be recognized by the immune system and can induce a T cell-targeted response. These TAAs are proteinase 3, the receptor for hyaluronan mediated motility and Wilms tumor protein (6). Moreover, various cell types such as αβ and γδ T cells, and NKT and NK cells have proven to be functional against AML cells together with a series of effector molecules such as perforin and tumor necrosis factor-related apoptosis-inducing ligand, but also IFN-γ, IFN type I, and IL-12 (7–9). Based on these premises, since the immune system is activated against leukemia cells, the possibility to harness immunity against AML to obtain a durable leukemia-specific immune response should not be underestimated in the clinical management of AML patients.

## Immunogenic Cancer Cell Death

In recent years, the concept of two principal forms of cell death which can promote tolerance (e.g., apoptosis) or immunity (e.g., necrosis) was first challenged and then surpassed, and a number of factors determining whether a death is tolerogenic or immunogenic was identified (10). An example is represented by the type of cell death, induced by therapeutics such as ionizing radiations or cytotoxic chemotherapy. Selected antineoplastic agents, in particular ionizing irradiation, anthracyclines, oxaliplatin, cyclophosphamide, mitoxantrone, and others (11–13), are able to induce a type of cell death which is apoptotic in morphology, but caspase-dependent and highly efficient in immune response induction without any adjuvant (14, 15). Such a death process, called immunogenic cell death (ICD), was introduced for the first time 10 years ago (16–20). During this process, the TAAs are released from dying tumor cells together with some factors, known as damage-associated molecular pattern molecules (DAMPs), generated in cell-stress conditions, hypoxia or nutrient depletion, which bind receptors expressed on immune cell surfaces, thus stimulating innate immune responses. In this context, specialized antigen-presenting cells, i.e., dendritic cells (DCs), play a crucial role in efficiently priming TAA-specific T cells (21). Subsequent studies have identified various mechanisms of the ICD process, and also highlighted the importance of the host capacity to detect the ICD events and induce a therapeutically relevant immune response against dying cells (16, 17, 20, 22, 23).

## ICD Events and Immune System Activation

ICD biology has been actively studied over the last 10 years. Very schematically, ICD is represented by the coordinated emission of a series of DAMPs (24–29), including the translocation of the endoplasmic reticulum (ER) chaperones such as calreticulin (CRT) and heat shock proteins 70 and 90 (HSP70 and 90) on cell surface, the adenosine triphosphate (ATP) active secretion, the non-histone chromatin-binding protein high mobility group box 1 (HMGB1) release from nucleus in extracellular milieu (30–37) and finally, the release of immunostimulatory cytokines, such as type I IFN (38).

In the early phase, CRT translocates from the ER to the outer leaflet of the plasma membrane, thus initiating the apoptotic caspase-dependent process. Simultaneously, the HSP70 and HSP90 bind TAAs and contribute to stimulate DC maturation. During the tardive post-apoptotic phase, pro-inflammatory factor HMGB1, which binds toll-like receptor 4 (TLR 4) on DCs, is released from the nucleus in the extracellular space. Finally, autophagy-dependent active secretion of ATP, which binds purinergic receptors (P2Rs) on DCs, promotes DC recruitment, survival and differentiation (39, 40).

When emitted in the correct spatiotemporal context, these DAMPs recruit DCs in the proximity of ICD and activate them to engulf TAAs. As a consequence, DCs become fully matured and competent in skewing cytokine production toward immunostimulatory cytokines, like IL-1β, IL-12p70, and IL-6, in spite of immunosuppressive cytokines, such as IL-10 (30, 34, 36), this process being strictly required for the adequate polarization of IFN-γ producing CD8^+^ T cells. The activation of APCs generally proceeds in two sequential phases, i.e., recruitment of T cells followed by their activation into IL-17- secreting γδ T cells, αβ Th1 T cells (IFN-γ secreting CD4^+^ T cells), and αβ cytotoxic T cells (IFN-γ secreting CD8^+^ T cells) (27, 31, 34). The latter are not only capable of mediating direct anti-tumor effects, but also underlie the establishment of host-protective immunological memory. Importantly, CRT exposure, HMGB1 release, and ATP secretion are indispensable for ICD. Indeed, the absence of just one of these ICD hallmarks cancels out the efficacy of ICD in mouse model (41).

### Early ICD Events

#### CRT Translocation

CRT can be localized in the cytoplasm, on the cell membrane or in the extracellular matrix, operating in both extra and intracellular space. Inside the ER, CRT plays an essential role in the regulation of intracellular Ca^2+^ homeostasis and storage, thus participating in a large variety of Ca^2+^-dependent signal transduction mechanisms. Moreover, CRT is involved in CRT/calnexin cycles where, interacting with calnexin and 57-kDa protein ER (ERp57), it ensures the correct folding of newly synthesized proteins and glycoproteins. In this context, CRT is fundamental also for assembly of major histocompatibility complex (MHC) molecules, which are essential for class I antigens presentation (42). Exposure to ICD inducers like anthracyclines, oxaliplatin, or ionizing radiation is able to induce translocation of CRT/ERp57 complex to the cell surface. Although the whole process underlying CRT protein exposure is far from being fully elucidated, three steps have been clearly identified: ER stress induction, apoptosis and translocation. Initially, stress response induction causes activation of reticulum PERK serin/treonin kinase which phosphorylates the eukaryotic translation initiation factor 2α (eIF2α) following the partial caspase-8 activation, the caspase-8-mediated cleavage of BAP31 and structural activation of pro-apoptotic proteins BAX and BAK. Finally, the translocation process predicts exocytosis through a SNARE-dependent mechanism in which CRT and ERp57 are transported inside vesicles to the outer plasma membrane leaflet of Golgi apparatus (43, 44).

CRT exposure represents an “*eat me signal*” for DCs, the crucial component of immune system activation by chemotherapy (45). CRT initiates phagocytosis of apoptotic cells binding to the CD91 receptor (known as LDL-correlate receptor protein; LRP) on phagocytic cells. The presence of a CRT specific receptor on DCs and its activation are essential for immunogenicity of tumor cell death. Interestingly, CRT translocation also occurs in viable malignant cells (46), suggesting that apoptosis may not be necessarily required for CRT translocation, and that “ER stress” induction can be sufficient to promote its cell surface expression (47).

#### HSP Exposure

HSPs play an important role as chaperones ensuring the correct folding of newly synthesized proteins or damaged proteins as a consequence of cellular stress and preventing their aggregation. However, HSPs can have a double role based on cellular localization. In the case of HSP 90, the intracellular localization determines a cytoprotective function responsible for addressing of damaged proteins toward proteasome degradation, thus maintaining protein homeostasis (48). On the contrary, when located inside the cells, HSP70 interacts with various components of apoptotic machinery at both pre- and post-mitochondrial level, thus preventing an inappropriate ICD induction caused by stress-induced cell damage. Importantly, the HSPs can translocate to the outer plasma membrane leaflet (HSP 70) or can be released into the extracellular space (HSPs 70 and 90). HSP exposure/release from cells that underwent ER stress represents one of the distinctive factors of chemotherapy-induced ICD (48).

HSPs can potentiate immunogenicity in different ways. On one hand, when present on the cell surface of tumor cells they can improve the recognition and up-take of dying cells by DCs. On the other hand, TAAs derived from dying tumor cells can bind HSPs, thus enhancing efficient antigen presentation. HSP-antigen complex recognition is mediated by TLR4, which facilities intracellular processing and presentation of TAAs (48).

Collectively, these findings indicate that the presence of HSPs on dying tumor cells is critical for tumor cell recognition by DCs, full DC maturation and, thus, for the induction of a tumor-specific immune response.

### Late ICD Events

#### Release of HMGB1 From the Nucleus

HMGB1 is a nuclear protein, which participates in the folding of DNA in the chromatin structure, thus influencing transcription and other nuclear functions. In contrast to histones, which are part of nucleosomes, the interaction of HMGB1 with chromatin is rather loose, which means that HMGB1 can exit from the nucleus to the cytoplasm. Importantly, HMGB1 also acts as an extracellular signal molecule, DAMP, and can be released from cells by non-canonical secretion pathways or passively released through the permeabilized plasma membrane of dead cells (49). Indeed, after cellular stress, HMGB1 translocates to cytosol and is then released to the extracellular space. When it binds to specific receptors, together with other cytokines, HMGB1 can induce myeloid DC maturation by CD40, CD54, CD80, CD83, and MHC II upregulation (20).

Under certain circumstances, cells dying by apoptosis or autophagy can release HMGB1, as observed in the case of DNA damage induced by UV radiation or platinization, where HMGB1 is sequestrated in the nucleus and the ICD inducers, such as anthracyclines, and stimulate HMGB1 release in the late phase of apoptosis (20). HMGB1 released in the extracellular space binds mainly the TLR4 present on DCs, thus facilitating TAA processing and presentation through the inhibition of phagosome and lysosome fusion, and the prevention of early degradation and by allowing their transport to effector immune cells (48). Moreover, it has been demonstrated that HMGB1 released during tumor cell necrosis induces not only DC maturation, but also secretion of IL-12 by DCs and IFN-γ by T cells acting as a potent stimulus for polarization of Th1 response (20).

#### ATP Extracellular Release

One of the most distinctive features of ICD is represented by the active extracellular release of ATP from dying cells during the tardive phase of apoptosis. Normally located inside the cells, ATP is considered the most important factor for bioenergetics, connecting anabolism and catabolism, with a well-established crucial role in some important processes, such as cellular motility, phosphorylation, and active transport. By specifically testing which of the P2Rs is involved, as well as the type or the optimal concentration of released nucleotides within the extracellular environment, various and seminal studies demonstrated the role of extracellular nucleotides in the regulation of cell proliferation, migration, and death (50, 51).

While different mechanisms of ATP release are known, the elevated release of ATP during ICD induced by chemotherapy principally depends on the induction of the autophagy process. Autophagy is a multistep process that involves cytoplasmic material sequestration within double-membraned organelles, autophagosomes, and their fusion with lysosomes (52). Importantly, besides its role as a DAMP molecule, extracellular ATP represents a strong “find me” signal which facilitates DC recruitment in sites of massive apoptosis (53). DC recruitment in tumor sites is mediated by P2Y2 receptors (54), whereas activation of P2Y11 receptors on monocytes and DCs induces their maturation (55). Once recruited, naive immune cells need activation signals to increase their anti-tumoral activity. It has recently been demonstrated that P2XRs are essential for the immune response induced by chemotherapy. ATP released from dying cells binds to the P2X7 receptor present on DCs, thus determining assembly and activation of inflammasome NOD-like Receptor protein 3 (NLRP3)/ASC/caspasi-1 driving the IL-1β secretion. The IL-1β is fundamental for adequate recruitment of γδ T lymphocytes secreting IL-17 and cytotoxic CD8^+^IFN-γ^+^ tumor-specific T lymphocyte generation (56, 57).

## ICD in Solid Tumors and Leukemias

Recent data support the role of chemotherapy in activating the immune response both in solid tumors (9, 11, 12, 44, 58) and, recently, in leukemias (34, 47, 59–62), with important therapeutical implications.

For a long time, the immune system was considered as a passive bystander of cancers, until the antineoplastic potential of new drugs in immunodeficient murine models was tested. Accumulating preclinical evidence has indicated that murine tumors respond more efficiently to therapies in immunocompetent individuals than in immunodeficient hosts (63, 64), suggesting an important role of the immune system mediating the chemotherapy effects. Several anticancer agents, for example anthracyclines in colorectal cancer (9), fibrosarcomas (57), and methylcholanthrene-induced tumors (65), cyclophosphamide in mesotheliomas (66), oxaliplatin in colorectal carcinomas and fibrosarcomas (12, 31), and cisplatin in combination with digoxin in fibrosarcomas (67) were tested and proven to activate the immune system, which ultimately and crucially contributes to the clinical response of cancers to chemotherapy treatment.

Regarding chemotherapeutic drugs, it is very important to differentiate between the direct immunogenic effects that such therapeutic regimens exert on tumor cells, and the capacity of chemotherapy-treated tumor cells to interact with the host immune system, resulting in reactivation of immune effectors, or in relief of immune-suppressive mechanisms. Three principal ways in which antineoplastic agents may stimulate the immune system were defined by Zitvogel et al.: increasing the antigenicity of cancer cells (increased TAA expression or presentation) (i); increasing the immunogenicity of cancer cells (DAMP production and release) (ii) and increasing the susceptibility of cancer cells (better recognition and killing of cancer cells by immune effectors) (iii) (68). As for the enhancement of antigenicity, cyclophosphamide, oxaliplatin and γ irradiation have been shown to increase MHC I molecule expression by cancer cells (69, 70), whereas γ irradiation, 5-fluorouracil or vemurafenib increased TAA expression (69, 71, 72). Regarding immunogenicity, anthracyclines, oxaliplatin, mafosfamide, bortezomib, and some other types of chemotherapy agents are effective in inducing CRT and HSP exposure (11, 12, 32, 47, 59, 61), as well as ATP secretion (31, 52, 55) and HMGB1 release (12, 73) from various tumor cells including leukemias. Finally, to increase the susceptibility of cancer cells, different anticancer agents including anthracyclines have been shown to sensitize murine tumor cells to the cytotoxic function of CTLs (74). Moreover, other pieces of evidence indicate that chemotherapy favors breast cancer cell infiltration by myeloid and granzyme B-expressing cells, while increasing the intra-tumoral CD8^+^ and CD4^+^ T cell ratio (75). Taken together, accumulating evidence suggests that, in some settings, tumor-specific immune responses induced during chemotherapy drive the destiny of cancer patients (76, 77).

For hematological malignancies, recent studies have demonstrated that anthracyclines trigger ICD *in vitro* and in murine models (78) including AML (34, 47). In particular, in AML patients, following anthracycline administration, CRT translocates from the nucleus to the leukemia cell surface. Indeed, Fredly et al. has demonstrated that CRT is exposed by apoptotic primary human AML cells in 65% of tested patients and that, *in vitro*, cultured AML cells showed spontaneous release of HSP70 and 90 (62). Of note, similarly to solid tumors, including neuroblastoma, non-small cell lung carcinoma, ovarian cancer, and colorectal carcinoma, where CRT exposure has been shown to be an important prognostic factor (79–81), CRT exposure by AML cells has been recently correlated by Fucikova et al. with a strong anticancer immune response, improving the clinical outcome of AML patients (59, 60). Surprisingly, these authors have found that DAMP emission from AML may also be chemotherapy-independent. In particular, 82% of AML patients exhibited positivity for CRT expression prior to treatment and a similar pattern was observed also for HSP exposure and HMGB1 release, thus suggesting that DAMP production may represent an intrinsic feature of some types of AML, which make them more prone to interact with the immune system. Indeed, CRT exposure was associated with enhanced anti-leukemia immune response and better prognosis. Transcriptional and phenotyping signature analysis in patients with AML has revealed robust vs. weak CRT exposure on blasts. Moreover, AML patients are prognostically divided into two groups based on the median percentage of circulating ecto-CRT, HSP70, or HSP90 positive cells, thus revealing that ICD-associated DAMPs correlate with improved disease outcome (60). CRT exposure on malignant blasts predicts a cellular anticancer immune response in patients with AML (61).

## Mechanisms of Immunologic Tolerance in AML

Along with the well-known cell-intrinsic mechanisms by which leukemic cells can develop drug resistance, which leads to enhanced proliferation and survival, the role of cell-extrinsic factors, partly derived from AML bone marrow, the immunosuppressive microenvironment has recently been investigated (82). It is known that both the innate and adaptive immune systems are deeply affected and profoundly deregulated by the interaction with leukemia cells. This happens as a result of several different immunosuppressive mechanisms, which, in turn, may lead to the escape of leukemia cells from the natural immunological control (82). Many of these regulatory mechanisms seem to be shared by solid tumors and hematology neoplasms including over-expression of inhibitory check-point receptors on T cells and their ligands on AML cells or DCs such as PD-1/PD-L1, CTLA-4/CD80/CD86, Tim-3/galectine-9 (gal-9), and Lag-3/MHCII, enzymes as IDO and induction of immunoregulatory populations expansion as Tregs (83–85). The most known suppressive mechanism in AML is the up-regulation of IDO1 expression on leukemia cells. IDO1 is responsible for catalyzing the initial rate-limiting step of tryptophan degradation resulting in increased final product kynurenines. The kynurenines have suppressive properties and increase the conversion of CD4^+^25^−^ T cells into Tregs. In addition, their suppressive effect relies on the fact that they can reduce the activity of NK cells, DCs or proliferating T cells, in response to inflammation or infection (85, 86). The most known inhibitory pathways in AML are reported in Table 1.

**Table 1:** Inhibitory pathways in AML.

**Inhibitory pathway/check point**	**Physiological role**	**Role in AML**	**Clinical trials in AML**
PD-1/PD-L1 axes	**PD-1**—receptor of negative co-stimulation. Ligands: **PD-L1** and **PD-L2**. **PD-1/PD-L1 axes**—control of normal immune responses, involved in periphery tolerance, autoimmunity regulation, allergy, infections, and antitumor immunity (87).	**PD-1/PD-L signaling**—dampening of anti-leukemic immunity in AML. **PD-L1** and **PD-L2** expression on human AML cells at diagnosis and relapse (88). **Blocking of PD-1/PD-L1 axis**—increase of anti-leukemia immune response and prevention of AML progression in murine model (83, 84, 89).	**1. Anti-PD-1** **Nivolumab:** NCT02275533, NCT02397720, NCT02532231, NCT03092674, NCT02464657, NCT02275533, NCT03066648 **Pembrolizumab:** NCT02708641, NCT02845297, NCT02996474, NCT02771197, NCT02768792 **Avelumab:** NCT02953561 **2. Anti-PD-L1** **Durvalumab:** NCT02775903 **Atezolizumab:** NCT02892318, NCT03154827
CTLA-4	**CTLA-4**—receptor of negative co-stimulation. Ligands: **CD80** and **CD86**. **CTLA-4/CD80/CD86 pathway**—regulation of T cell response (83).	**CTLA-4/CD80/CD86**—hampering T cell immunity against hematological malignancies (83) and modulating immune responses in AML (90). **Blocking of CTLA-4 pathway—**increase of anti-leukemia T-cell immune response translated in prolonged tumor regression (91, 92).	**1. Anti-CTLA-4** **Ipilimumab:** NCT00039091, NCT02890329, NCT02397720
CD200R/CD200	**CD200R**—inhibitory receptor. Ligand: **CD200**. **CD200-CD200R signaling—**down-regulation of immune responses preventing inflammation and immune pathology (83).	**CD200R/CD200**—immunosuppressive signal transmission, macrophages inhibition, Tregs induction and tumor-specific T cells inhibition (93).Expression of **CD200** on human AML cells (94)—worse overall survival of some AML subsets (83). **Blocking of CD200—**enhanced cytotoxicity of NK cells, restored proliferative capacity of T cells, dampens tumor-reactive immune responses (95), but also favors tumor progression due to enhanced pro-tumorigenic inflammation (96).	**1. Anti-CD200** **Samalizumab:** NCT03013998
Lag-3	**Lag-3** receptor of negative co-stimulation. Ligand: **MHC II**. **Lag-3/MHCII signaling** - tolerance maintenance (83, 97).	**Lag-3** signaling-suppression of CTL activity in tumors (97, 98). **Blocking of PD-L1**, **CTLA-4** and **Lag-3**—effective and enduring immunotherapy for disseminated leukemia in murine model (98).	To date—no clinical trials available
Tim-3	**Tim-3**—receptor of negative co-stimulation. Ligands: **gal-9/HMGB1/phosphatidyl serin**. **Tim-3/gal-9 signaling**—regulation of T-cell tolerance (83).	**Tim-3** released by AML**—**reduce ability of T cells to secrete IL-2 required for NK and CTLs activation (99). **TIM-3** and **PD-1** co-expression on T cells was associated with AML progression in mouse and human (7) and with relapse in AML patients after allo-SCT (100). **TIM-3**—overexpression on AML (stem) cells (101) and T cells of newly diagnosed AML -(102). **Blocking of TIM-3 and PD-1**—reduced tumor burden and improved survival in AML murine model (7).	**1. AntiPD-1** **+** **TIM-3** **PDR001+MBG453+** **Decitabine:** NCT03066648
IDO and Tregs	**IDO** –immunosuppressive and tolerogenic enzyme responsible for tryptophan degradation in kynurenines with subsequent T cell inhibition and **Tregs** expansion. **Tregs—**role in maternal tolerance, autoimmune disease regulation, suppression of transplant rejection (85).	**IDO** signaling—Tregs induced by IDO-expressing leukemic DCs impair leukemia-specific CTL (103).Increased **IDO** activity—lower CR rates and shorter OS in AML (103–105). **Blocking of IDO—**effective immune response in AML *in vitro* (103–106).	**1. Anti-IDO** **Epacadostat:** NCT03444649

*Different inhibitory pathways and their role in both physiological and AML contexts are correlated with clinical trials ongoing for specific pathways*.

## Tolerogenic Mechanisms During ICD

In the ICD scenario, some recent reports indicate that, along with the activation of the immune system, a wide variety of tolerogenic mechanisms is also induced, mostly resulting in Tregs induction (53, 107, 108). In particular, Tregs induction after immunogenic chemotherapy was observed in some solid tumors. Bugaut et al. demonstrated that bleomycin, an anti-tumor antibiotic glycopeptide produced by bacterium Streptomyces and used for the treatment of cancer testis and Hodgkin disease, induces both ICD resulting in anti-tumor CD8^+^ T cell response and Tregs accumulation *in vivo*. Specifically, bleomycin induces expansion of Foxp3^+^ Tregs via its capacity to induce transforming growth factor beta (TGF-β) secretion by tumor cells. Accordingly, Tregs or TGF-β depletion dramatically potentiates the antitumor effect of bleomycin. Based on these premises, it is conceivable that in order to fully exploit the activatory capacity of immune response by immunogenic chemotherapy, it may be fundamental to concomitantly block chemotherapy-driven Tregs induction.

Similarly to solid tumors, also in AML it is well-known that inducing a suppressive microenvironment by expanding Tregs may hamper the anti-leukemia immune response (103, 109). Interestingly, early lymphocyte recovery in 20 patients undergoing induction chemotherapy for newly diagnosed AML indicated that recovering T cells were predominantly activated Tregs with suppressive activity. Despite an initial burst of thymopoiesis, most recovering Tregs were of peripheral origin and showed marked oligoclonal skewing, suggesting that their peripheral expansion was antigen-driven (108). Wang et al. too demonstrated a rapid turnover of Tregs in AML patients after chemotherapy compared to healthy controls (110). Together, these findings suggest an important role of Tregs induction after chemotherapy in AML.

We recently investigated the mechanisms underlying the effects of chemotherapy on Tregs induction. In particular, we focused on the tolerogenic role of leukemia-infiltrating DCs after chemotherapy. Our *in vitro* and *in vivo* data demonstrate that during ICD a population of DCs expressing IDO1 is responsible for the induction of Tregs (106). In particular, we demonstrated that ATP released from chemotherapy-treated AML cells is responsible for IDO1 up-regulation on DCs through the P2X7 receptor and consequent Tregs enrichment, resulting in the establishment of an immune suppressive microenvironment. Moreover, the analysis of the T-cell composition emerging in AML patients after induction chemotherapy revealed an enrichment and activation of the most suppressive Tregs-subpopulation expressing FOXP-3, CTLA-4, CD39, PD-1, and Ki-67 (106). These results demonstrated that ATP released from chemotherapy-treated dying leukemic cells during ICD has a role in the induction of the immune suppressive microenvironment, which comprises Tregs and IDO1-expressing DCs (106).

**Figure 1:**
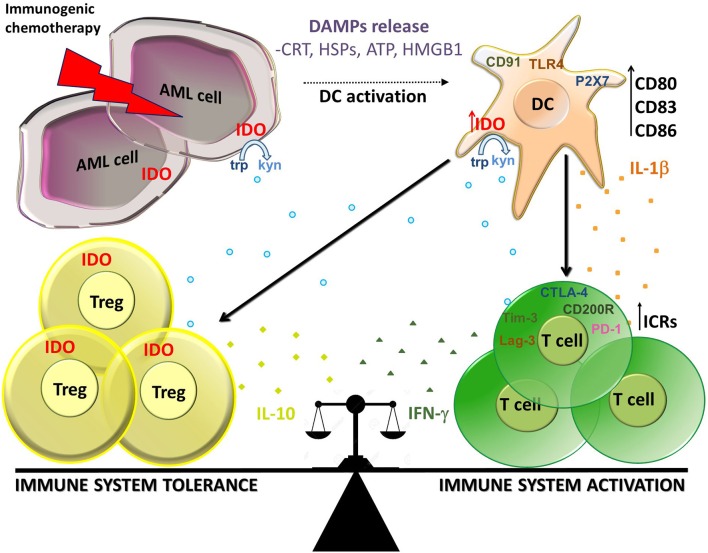
Balance between immune activation and tolerance during ICD in AML. Immunogenic chemotherapy causes the release of DAMPs (CRT, HSPs, ATP, and HMGB1) which bind to receptors on DCs as CD91, TLR4, and P2X7. DCs up-regulate maturation markers (CD80, CD86, and CD83) and produce IL-1β resulting in activation of T cells producing IFN-γ At the same time, DCs up-regulate IDO1 which is responsible for the production of kynurenines which in turn stimulate induction of Tregs producing IL-10 and inhibit effector T cells. IDO1 is expressed also on AML cells and Treg cells, thus participating to the suppressive local milleu. Immune check points receptors (ICRs) as PD-1, Tim-3, Lag-3, CD200R, and CTLA-4 can contribute to the cell composition of tumor microenvironment. In this context, IDO1 seems to play a key role in the balance between immune system activation and tolerance in AML during ICD.

Taken together, these findings suggest that IDO and related downstream pathways resulting in Tregs induction may play an important regulatory role in the choice between tolerance or immunity in response to dying tumor cells (Figure 1) and are in line with other recent studies which use preclinical models of self-tolerance and autoimmunity (85). In this scenario, chemotherapy-induced ICD can prompt both immune tolerance and activation through the same mechanisms, and the balance between these phenomena can be fundamental for the final immune system response.

## Concluding Remarks

Some antineoplastic agents are capable of activating the immune system through the release of inflammatory signals from dying tumor cells. However, recent evidence indicates that chemotherapy may also provide the tumor microenvironment with a number of tolerogenic signals, mainly resulting in Tregs induction, which negatively influence immune response activation. Interestingly, the same mechanisms leading to immune activation are suggested to be also responsible for tolerance induction. Then, to fully exploit the immunogenic potential of chemotherapy, it is necessary to concomitantly act by inhibiting tolerance induction. Indeed, early clinical studies are testing the safety and early efficacy of new immunological agents contrasting tolerogenic mechanisms, such as IDO1 and immune checkpoint inhibitors, in combination with immunogenic chemotherapy.

Although this dual process is relevant in many tumors, it is particularly important in the setting of AML, where chemotherapy still constitutes the most powerful and curative therapeutical tool for most patients. For these reasons, in the AML field these studies will help in better understanding the biology of ICD, including the critical balance between activation and tolerance, thus providing the rationale for moving another step forward for an integrated immunological approach to AML therapy.

## Author Contributions

DO wrote and revised the manuscript and was the major contributor. ML wrote and revised sections of the manuscript. FL collected the related papers. SA, GV, MC, and AI participated in the design of the review and helped to draft and revise the manuscript. AC wrote sections of the manuscript, participated in the design of the review, and helped to draft and revise the manuscript. All authors read and approved the final manuscript.

### Conflict of Interest

The authors declare that the research was conducted in the absence of any commercial or financial relationships that could be construed as a potential conflict of interest.
